# Supplementary material for: General Practitioner’s Knowledge about Bariatric Surgery Is Associated with Referral Practice to Bariatric Surgery Centers
Source: Int J Environ Res Public Health. 2021 Sep 24;18(19):10055. doi: 10.3390/ijerph181910055 (PMC8508327; doi:10.3390/ijerph181910055)
Supplement: Supplementary file 1 [file ijerph-18-10055-s001.zip › ijerph-1394880-supplementary.pdf]

UNIKLINIK RWTHAACHEN
Lehrgebiet für Allgemeinmedizin
Klinik für Allgemein-, Viszeral- und Transplantationschirurgie

Personal information

Please send the questionnaire via FAX to:
0241-80-82458

Age:

Gender: m ☐ f ☐

Height [m]:

Weight [kg]:

General treatment of obese patients

How frequently do you calculate your patients' BMI?
(1) never
(2) almost never
(3) only visibly overweight patients' BMI
(4) almost always
(5) always

How confident are you in approaching obese patients concerning their weight and related risks?
(1) insecure
(2) mostly insecure
(3) neutral
(4) mostly confident
(5) confident

How confident are you in educating obese patients regarding their obesity, if they ask you?
(1) insecure
(2) mostly insecure
(3) neutral
(4) mostly confident
(5) confident

How many patients did you provide with follow-up care after their weight loss surgery?
(1) 0
(2) 1-2
(3) 3-4
(4) 5
(5) more than 5

Stigmatization

„Obese people are lazy“
(1) totally agree
(2) mostly agree
(3) neutral
(4) mostly disagree
(5) totally disagree

„Obese people have no self-control“
(1) totally agree
(2) mostly agree
(3) neutral
(4) mostly disagree
(5) totally disagree

„Obese people are unattractive“
(1) totally agree
(2) mostly agree
(3) neutral
(4) mostly disagree
(5) totally disagree

„Obese people are self-responsible for their overweight“
(1) totally agree
(2) mostly agree
(3) neutral
(4) mostly disagree
(5) totally disagree

„Obese people have no self-confidence“
(1) totally agree
(2) mostly agree
(3) neutral
(4) mostly disagree
(5) totally disagree

Weight loss surgery and multimodal weight loss program

How many patients have you educated on the possibility of weight loss surgery in the last six months?
(1) 0
(2) 1-2
(3) 3-4
(4) 5
(5) more than 5

How many patients have you referred to a bariatric surgery center?
(1) 0
(2) 1-2
(3) 3-4
(4) 5
(5) more than 5

Are you familiar with the regarded criteria for undergoing weight loss surgery?
(1) unfamiliar
(2) mostly unfamiliar
(3) neutral
(4) mostly familiar
(5) familiar

Do you know surgical procedures such as Roux-en Y gastric bypass and sleeve gastrectomy?
(1) totally disagree
(2) mostly disagree
(3) neutral
(4) mostly agree
(5) totally agree

„It is too easy for obese people to lose weight with weight loss surgery."
(1) totally agree
(2) mostly agree
(3) neutral
(4) mostly disagree
(5) totally disagree

In my opinion, weight loss surgery is a useful treating tool for obesity and related comorbidities.
(1) totally disagree
(2) mostly disagree
(3) neutral
(4) mostly agree
(5) totally agree

Are you familiar with the multimodal weight loss program for treating obese patients?
(1) unfamiliar
(2) mostly unfamiliar
(3) neutral
(4) mostly familiar
(5) familiar
